# Emulation of the control cohort of a randomized controlled trial in pediatric kidney transplantation with Real-World Data from the CERTAIN Registry

**DOI:** 10.1007/s00467-022-05777-x

**Published:** 2022-10-20

**Authors:** Christian Patry, Lukas D. Sauer, Anja Sander, Kai Krupka, Alexander Fichtner, Jolanda Brezinski, Yvonne Geissbühler, Elodie Aubrun, Anna Grinienko, Luca Dello Strologo, Dieter Haffner, Jun Oh, Ryszard Grenda, Lars Pape, Rezan Topaloğlu, Lutz T. Weber, Antonia Bouts, Jon Jin Kim, Agnieszka Prytula, Jens König, Mohan Shenoy, Britta Höcker, Burkhard Tönshoff

**Affiliations:** 1 Department of Pediatrics I, University Children’s Hospital Heidelberg, Heidelberg, Germany; 2 Institute of Medical Biometry, University of Heidelberg, Heidelberg, Germany; 3 Novartis Pharma AG, Basel, Switzerland; 4 Novartis Pharmaceuticals, East Hanover, NJ USA; 5 Renal Transplant Unit, Bambino Gesù Children’s Hospital, Pediatric subspecialities, Rome, Italy; 6 Department of Pediatric Kidney, Liver and Metabolic Diseases, Hannover Medical School, Hannover, Germany; 7 Pediatric Nephrology, University Hospital Hamburg, Hamburg, Germany; 8 Department of Nephrology, Kidney Transplantation and Hypertension, Children’s Memorial Health Institute, Warsaw, Poland; 9 Clinic for Paediatrics III, Essen University Hospital, Essen, Germany; 10 Department of Pediatric Nephrology, School of Medicine, Hacettepe University, Ankara, Turkey; 11 Pediatric Nephrology, Children’s and Adolescents’ Hospital, University Hospital Cologne, Medical Faculty University of Cologne, Cologne, Germany; 12 Department of Pediatric Nephrology, Amsterdam University Medical Center, Emma Children’s Hospital, Amsterdam, The Netherlands; 13 Department of Paediatric Nephrology, Nottingham University Hospital, Nottingham, UK; 14 Pediatric Nephrology and Rheumatology Department, Ghent University Hospital, Ghent, Belgium; 15 Department of General Pediatrics, University Children’s Hospital, Munster, Germany; 16 Paediatric Nephrology, Royal Manchester Children’s Hospital, Manchester, UK

**Keywords:** Real-World Data, Emulated cohorts, Clinical trial design, Pediatric kidney transplantation

## Abstract

**Background:**

Randomized controlled trials in pediatric kidney transplantation are hampered by low incidence and prevalence of kidney failure in children. Real-World Data from patient registries could facilitate the conduct of clinical trials by substituting a control cohort. However, the emulation of a control cohort by registry data in pediatric kidney transplantation has not been investigated so far.

**Methods:**

In this multicenter comparative analysis, we emulated the control cohort (*n* = 54) of an RCT in pediatric kidney transplant patients (CRADLE trial; ClinicalTrials.gov NCT01544491) with data derived from the Cooperative European Paediatric Renal Transplant Initiative (CERTAIN) registry, using the same inclusion and exclusion criteria (CERTAIN cohort, *n* = 554).

**Results:**

Most baseline patient and transplant characteristics were well comparable between both cohorts. At year 1 posttransplant, a composite efficacy failure end point comprising biopsy-proven acute rejection, graft loss or death (5.8% ± 3.3% vs. 7.5% ± 1.1%, *P* = 0.33), and kidney function (72.5 ± 24.9 vs. 77.3 ± 24.2 mL/min/1.73 m^2^ *P* = 0.19) did not differ significantly between CRADLE and CERTAIN. Furthermore, the incidence and severity of BPAR (5.6% vs. 7.8%), the degree of proteinuria (20.2 ± 13.9 vs. 30.6 ± 58.4 g/mol, *P* = 0.15), and the key safety parameters such as occurrence of urinary tract infections (24.1% vs. 15.5%, *P* = 0.10) were well comparable.

**Conclusions:**

In conclusion, usage of Real-World Data from patient registries such as CERTAIN to emulate the control cohort of an RCT is feasible and could facilitate the conduct of clinical trials in pediatric kidney transplantation.

**Graphical abstract:**

**Figure:**
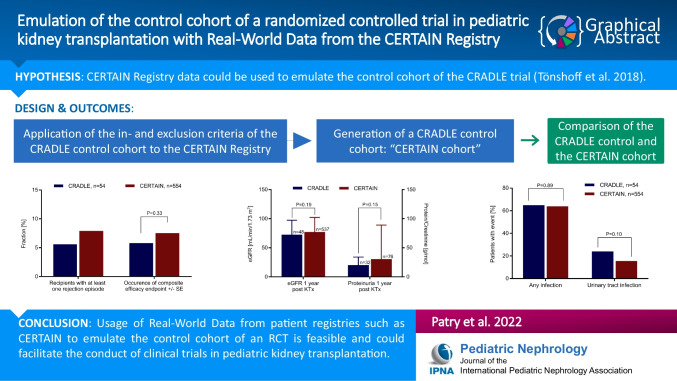
A higher resolution version of the Graphical abstract is available as Supplementary information

**Supplementary Information:**

The online version contains supplementary material available at 10.1007/s00467-022-05777-x.

## Introduction

The approval of new immunosuppressants in pediatric kidney transplantation requires evidence from randomized controlled clinical trials (RCTs). However, their conduct is time- and cost-intensive and subject to several limitations [1, 2]. Recruitment of study participants in patient populations with limited number of subjects is difficult, even in a multicenter setting. Studies with small numbers of participants may not capture rare adverse events [3]. Sources of Real-World Data could provide larger patient cohorts to clinical trials and thereby even capture rare adverse events [4]. Furthermore, RCTs may not adequately reflect the population in which the drugs will be used after their approval [5], because RCTs take place under idealized and thus artificial, controlled conditions. This might not necessarily do justice to the diversity of the populations the groups in RCTs are designed to represent [4, 6]. Also, the conduct of randomized trials is made difficult or even impossible in pandemic situations such as the current COVID-19 pandemic [7].

Real-World Evidence, which is derived from Real-World Data [8], could help to remedy the difficulties associated with the conduct of RCTs and possibly describe Real-World conditions even better [9].

According to the definition made by the FDA, Real-World Data are related to patient health and to the delivery of healthcare. They are derived from a variety of sources like electronic health care records or patient registries [8]. The generation of evidence from Real-World Data sources usually requires large amounts of data. Furthermore, experienced experts are needed to perform correct and valid analyses and — in addition — Real-World Data sources require much time for data quality management and maintenance [9]. However, they have many advantages over data derived from RCTs regarding the representation of Real-World conditions. For example, Real-World Data sources are more likely to capture rare adverse events, and they detect patient follow-up and variable treatment patterns resulting from every day clinical decision-making in the actual practice outside of the artificial construct of controlled studies [9]. Since the Century Cures Act of 2016, the US Food and Drug Administration (FDA) has been looking at whether and how to use observational data from Real-World Data sources [10]. The use of such Real-World Data in clinical study design and conduct is currently being discussed and investigated, including in clinical nephrology [10–12].

In pediatric nephrology and particularly pediatric kidney transplantation, clinical research is complicated by low patient incidence and prevalence. For example, in the USA in 2020, 715 children and adolescents under the age of 18 years underwent kidney transplantation [13]. In addition, randomization may not be feasible or ethical, for example, when an innovator drug has already proven superiority *vs.* standard of care therapy in adult kidney transplant recipients. The conduct of non-randomized, single arm trials with external Real-World Data as control could therefore be considered. External controls (e.g., historical controls) are a possible type of control arm in a comparative study [14]. Typically, the external control arm uses data from previous traditional clinical trials, but in some cases, Real-World Data have been used as the basis for external controls. Real-World Data derived from patient registries could be a valuable source to facilitate the conduct of clinical trials. However, the feasibility of such an approach in the field of pediatric kidney transplantation has not been studied so far. We therefore investigated the feasibility of using Real-World Data from the registry of the Cooperative European Paediatric Renal Transplant Initiative (CERTAIN) [15–17] which is characterized by high data granularity, quality, and completeness, to emulate the control cohort of an RCT in pediatric kidney transplantation, the CRADLE trial, to assess the outcome at year 1 posttransplant [18]. According to the definition by the FDA since the 21st Century Cures Act in 2016, the CERTAIN Registry fulfills all required criteria to be called a source of specific Real-World Data with regard to the population of pediatric kidney transplant recipients, because CERTAIN routinely assesses data relating to patient health status and relating to the delivery of health care [8].

## Materials and methods

In this multicenter comparative analysis, we emulated data of the control cohort of an RCT which compared standard tacrolimus with mycophenolate mofetil (MMF) and steroids (*n* = 54) to early conversion to everolimus with reduced tacrolimus and steroid elimination in pediatric kidney transplant patients (CRADLE trial) with data derived from the Cooperative European Paediatric Renal Transplant Initiative Registry (CERTAIN; www.certain-registry.eu), using the same inclusion and exclusion criteria as CRADLE (CERTAIN cohort).

### Data source

The web-based CERTAIN Registry was developed as a research platform and network for the highly specialized field of pediatric kidney transplantation with the aim to facilitate clinical research, quality assurance, and standardization of diagnostic and therapeutic approaches. CERTAIN includes pediatric kidney allograft recipients aged ≤ 21 years at time of transplantation. The registry’s dataset provides comprehensive information on kidney transplantation-related topics and pediatric-specific issues [15, 16]. Each contributing center's ethics committee had approved the CERTAIN Registry which is maintained in compliance with the principles of the Declaration of Helsinki and Good Clinical Practice guidelines. Written informed consent to participate in the registry was obtained from all parents or guardians, with assent from patients, if appropriate for their age. This study was designed, analyzed, and reported according to the STROBE (“STrengthening the Reporting of OBservational studies in Epidemiology”) guidelines (https://www.strobe-statement.org), which are published on behalf of the STROBE initiative. Adherence to these guidelines shall increase the transparency of published scientific research and results derived from observational trials [19]. The supplementary files provide further information regarding data completeness and data quality in CERTAIN in comparison with the CRADLE data (Supplemental Table 1). The supplementary files also provide the STROBE-Checklist for this study (Supplemental Table 2).

### Inclusion and exclusion criteria

**Fig. 1:**
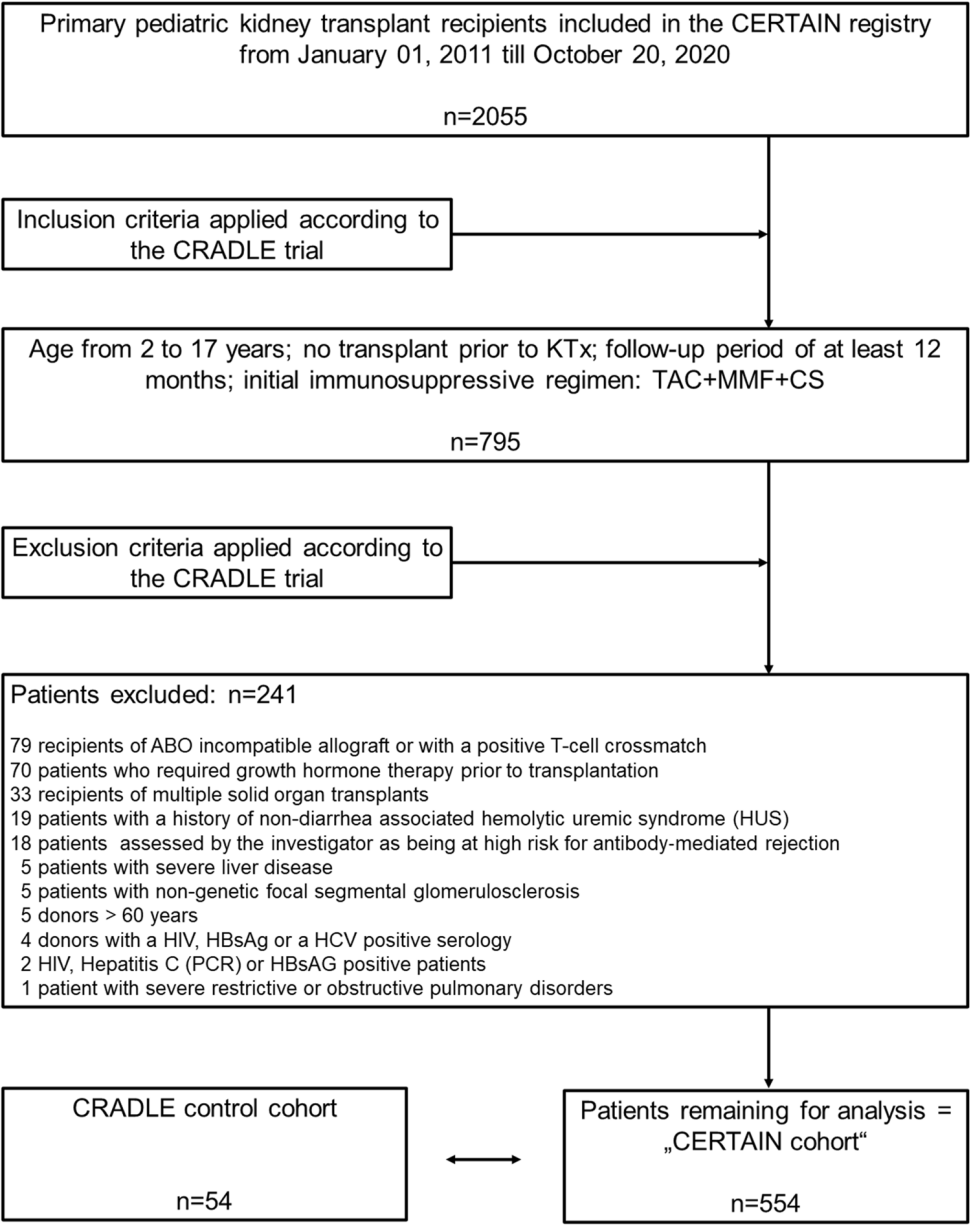
Study flow chart showing inclusion and exclusion criteria applied on the CERTAIN Registry data on October 20, 2020 to generate the CERTAIN cohort for comparison with the CRADLE control cohort. Exclusion criteria in the CRADLE trial, which were not applicable to the CERTAIN Registry, are shown Supplemental Table 2. CS, corticosteroids; KTx, kidney transplantation; MMF, mycophenolate mofetil; TAC, tacrolimus

The CRADLE trial was a 1‐year, multicenter, open‐label study; 106 children were randomized at 4 to 6 weeks after kidney transplantation to switch to everolimus with reduced tacrolimus exposure and steroid elimination from month 5 posttransplant or to continue standard tacrolimus with mycophenolate mofetil (MMF) and steroids [18]. Patients were eligible for enrolment to the run‐in phase of the study if they received a first or second kidney transplant at the age of ≥ 1 and < 18 years. For the key exclusion criteria in the CRADLE trial, see Supplemental Material. For emulation of the control cohort of the CRADLE trial, we considered all included patients in the CERTAIN Registry since initiation of data capture on January 1, 2011 until October 20, 2020 (*n* = 2055). We applied the same eligibility criteria as the CRADLE trial to emulate the control cohort of CRADLE as exactly as possible with data derived from CERTAIN (see Fig. 1 and Supplemental Material). A similar approach was recently successfully applied by Chen et al. in the context of investigating the feasibility of Real-World Data usage in Alzheimer’s disease research [20]. Supplemental Table 3 shows exclusion criteria applied in the CRADLE trial, which were not applicable to the CERTAIN Registry data.

### Endpoints

We compared data of the safety control population of the CRADLE trial, referred to as CRADLE control cohort [18], with the CERTAIN cohort regarding baseline patient and transplant characteristics and the following outcome data at month 12 posttransplant: graft loss, death, biopsy-proven acute rejection (BPAR), kidney transplant function (eGFR (mL/min/1.73 m^2^)) [21] and urinary protein-creatinine ratio (g/mol) [22], development of posttransplant lymphoproliferative disorders (PTLDs), number of patients with any infection and infections of the urinary tract, specific laboratory endpoints of interest, and anthropometric data. Height, weight, and body mass index data were converted to z-score values related to age- and sex-specific means and SD of respective reference populations [23–25].

### Sensitivity analysis with additional CERTAIN sub-cohorts

To increase the validity of data and results generated from the main analysis, we created two further and more specific sub-cohorts from the main CERTAIN cohort. One sub-cohort (CERTAIN sub-cohort “2012–2016”) was characterized by only including patients who received their kidney transplant in the same era (August 1, 2012–October 31, 2016) as in the CRADLE trial. The second sub-cohort (CERTAIN sub-cohort “basiliximab”) was designed to mirror the distribution pattern of induction therapy with basiliximab in the CRADLE control cohort (92.6% induction therapy with basiliximab). The CERTAIN sub-cohort “basiliximab” consisted of 169 patients from the main CERTAIN cohort who received basiliximab as induction therapy and 14 randomly selected patients from the main CERTAIN cohort, who did not receive induction therapy. We compared these two additional CERTAIN sub-cohorts to the CRADLE control cohort regarding the same baseline patient and transplant characteristics and outcome data at month 12 posttransplant as in the main analysis.

### Statistical analyses

For binary and categorical variables, numbers and percentages are given. For continuous variables, mean, standard deviation, median, quartile 1, quartile 3, minimum, and maximum are presented as appropriate. Incidence rates were calculated as the proportion of patients experiencing at least one event of interest in the first 12 months posttransplant. For continuous variables, a two-sided *t*-test was used. A pooled *t*-test was chosen whenever the equality of variance test returned *P* > 0.1, and a Satterthwaite *t*-test was chosen whenever the equality of variance test returned *P* < 0.1. To compare frequency variables, the chi-squared test was applied. Here, no *P* value was calculated when an expected frequency in the respective contingency table was less than 5. This is because the chi-squared approximation is poor for low expected frequencies. Fisher’s exact test, a common alternative, is systematically underpowered. For the comparison of the anthropometric *Z*-scores, only median and range were available in the CRADLE control cohort. Here, we chose to avoid the underpowered median test and instead reported distribution-free 95% confidence intervals as a measure of statistical uncertainty for the respective medians of the CERTAIN cohort. To compare efficacy endpoints, we used a composite efficacy failure endpoint comprising the following variables: first occurrence of BPAR, graft loss, or death, whichever event came first. The event-free survival rates at month 12 posttransplant with respect to the composite endpoint were calculated using the Kaplan–Meier estimator and its standard error. The respective *P* value for comparing these survival rates was calculated using a chi-squared test with one degree of freedom together with the clog test statistic X3 defined in Klein et al. [26]. This analysis is of exploratory nature, and all *P* values are to be interpreted in descriptive manner.

## Results

### Demographics and baseline characteristics

**Fig. 2:**
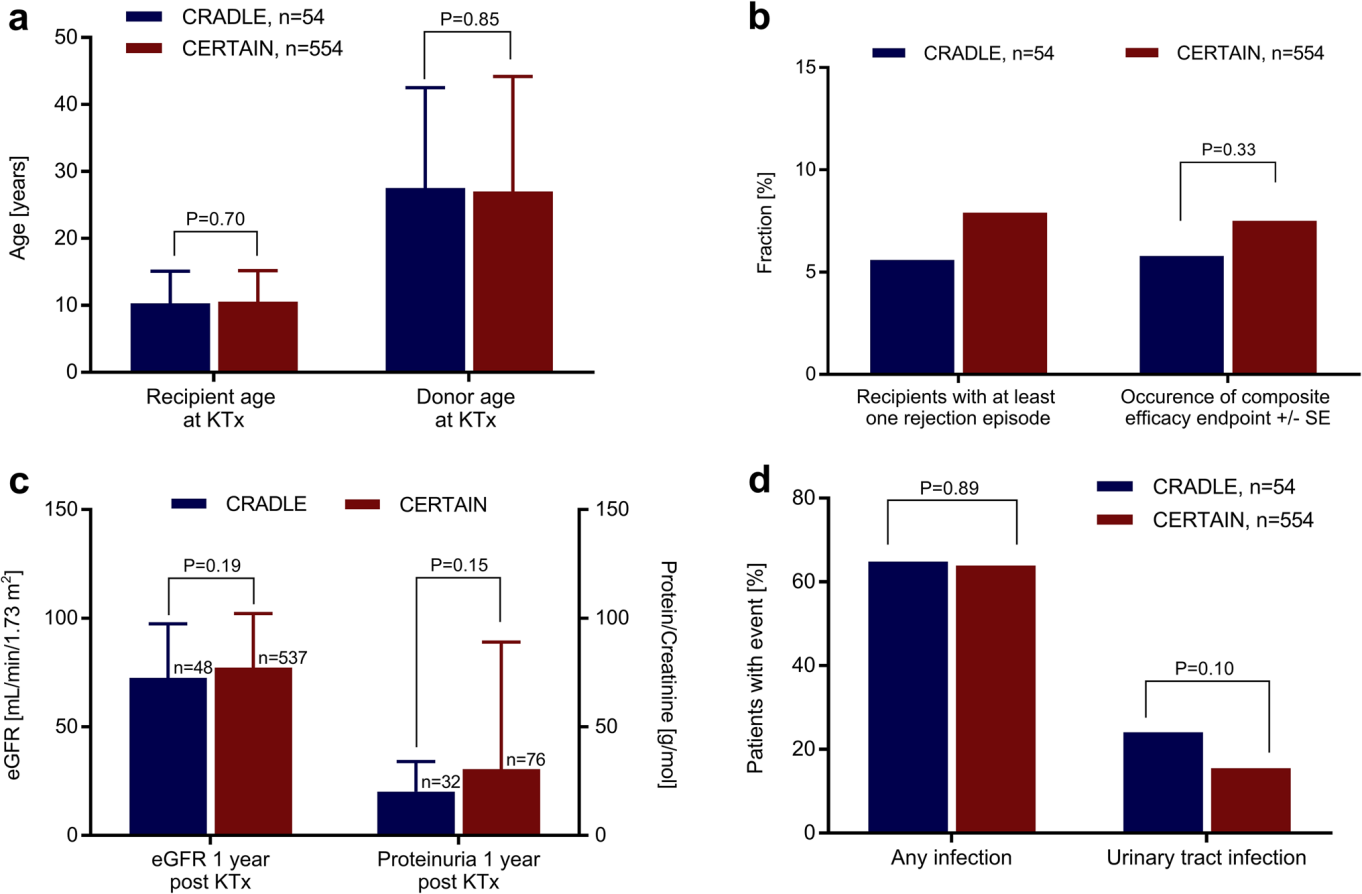
Comparisons of efficacy and safety endpoints between the CRADLE control cohort and the CERTAIN cohort. **a** Patient and donor age at time of kidney transplantation. **b** Patients with at least one biopsy-proven acute rejection episode and occurrence of the composite efficacy failure endpoint (BPAR, graft loss or death) at year 1 posttransplant (shown by Kaplan–Meier estimates). **c** eGFR and proteinuria at year 1 posttransplant. **d** Patients with at least one infection and with at least one urinary tract infection at year 1 posttransplant. eGFR, estimated glomerular filtration rate; KTx, kidney transplantation

The CERTAIN cohort comprised *n* = 554 patients, which is 26.9% of the total *n* = 2055 patients enlisted in CERTAIN at the time of data extraction (Fig. 1). Patients in the CERTAIN cohort received their kidney allograft between December 2002 and May 2019. Data from these patients were compared to the control cohort of the CRADLE trial (*n* = 54 patients); these patients received their kidney allograft between August 2012 and October 2016 [18]. Demographic data and baseline characteristics such as recipient age, male sex, race, donor age, the rate of kidney transplantations from living-related donors *vs.* deceased donors, and the incidence of delayed graft function were well comparable and did not differ significantly between the two cohorts (Table 1; Fig. 2a). There were slight differences regarding the type of primary kidney diseases (Table 1). The respective immunosuppressive regimen of the two cohorts at baseline and at year 1 posttransplant is shown in Table 1. One hundred seventy-three of 554 (31%) patients in the CERTAIN cohort received an induction therapy with either basiliximab (*n* = 169) or daclizumab (*n* = 4) compared to 50 of 54 (92.6%) patients in the CRADLE control cohort (*P* = 0.0001). While 47 of 54 (87.0%) patients in the CRADLE control cohort remained on the initial immunosuppressive regimen (tacrolimus, MMF, and steroids) during the first year posttransplant, this rate was lower in the CERTAIN cohort (379 of 554 [68.4%]; *P* = 0.0001).

**Table 1:** Patient and transplant characteristics

Patient characteristics	CRADLE control cohort (*n* = 54)	CERTAIN cohort (*n* = 554)	*P* value^a^
Age at KTx (years), mean ± SD	10.3 ± 4.8	10.6 ± 4.6	0.70
Male sex, *n* (%)	31 (57.4)	318 (57.4)	1.0
White/Caucasian race, *n* (%)	47 (87.0)	507 (91.5)	NA
Diabetes mellitus at randomization, *n* (%)	2 (3.7)	1 (0.2)	NA
Primary kidney diseases, *n* (%)			NA
Renal hypoplasia/dysplasia	19 (35.2)	117 (21.1)	
Obstructive uropathy/vesicoureteral reflux	7 (13.0)	98 (17.7)	
Polycystic kidney disease	5 (9.3)	77 (13.9)	
Glomerulonephritis/glomerular disease	12 (22.2)	98 (17.7)	
Systemic disorders including vasculitis	2 (3.7)	48 (8.7)	
Tubular disorder	3 (5.6)	10 (1.8)	
Other	6 (11.1)	106 (19.1)	
Immunosuppressants at KTx, *n* (%)			
Induction therapy (basiliximab or daclizumab)	50 (92.6)	173 (31.0)	0.0001
Tacrolimus + MMF + Steroids	54 (100)	554 (100)	1
Immunosuppressants at year 1 posttransplant, *n* (%)			
Tacrolimus + MMF + steroids	47 (87.0)^b^	379 (68.4)	0.0001
Kidney replacement therapy at time of KTx			0.064
None (preemptive KTx)	21 (38.9)	136 (24.6)	
Hemodialysis	16 (29.6)	184 (33.2)	
Peritoneal dialysis	17 (31.5)	234 (42.2)	
Donor-related data			
Age, mean ± SD	27.5 ± 15.0	27.0 ± 17.2	0.85
Living-related donor	23 (42.6)	182 (32.9)	0.15
Transplant function			
Delayed graft function, *n* (%)	2 (3.7)	38 (6.9)	NA

^a^*P* values of metric variables were calculated using the pooled *t*-test or the Satterthwaite *t*-test, depending on equality of variance. *P* values for frequency variables were calculated using the chi-squared test. No *P* values were calculated for frequency variables whenever the expected frequency in at least one of the cells was less than 5

^b^According to Supplemental Table 1 of the CRADLE trial^18^

*MMF* Mycophenolate mofetil, *KTx* kidney transplantation, *NA* not applicable (no *P* value was calculated, when the expected frequency in the respective contingency table was less than 5)

### Comparison of anthropometric data

The median height at baseline (prior to transplantation) and at year 1 posttransplant was slightly greater in CRADLE than in CERTAIN, while the respective changes between baseline and year 1 were comparable (Table 2). The median weight at baseline was greater in CRADLE than in CERTAIN, while data were comparable at year 1 posttransplant. The change of weight between baseline and year 1 was smaller in CRADLE than in CERTAIN (Table 2). Median BMI at baseline was higher in CRADLE than in CERTAIN and comparable at year 1 posttransplant. The change of BMI between baseline and at year 1 posttransplant was smaller in CRADLE than in CERTAIN (Table 2).

**Table 2:** Anthropometric data at baseline and year 1 posttransplant

*Z*-scores, median (range)	CRADLE control cohort *n* = 54	CERTAIN cohort Baseline, *n* = 554 Year 1, *n* = 537
Height		
Baseline	− 1.5 (− 4.5 to 2.2)	− 1.7 (− 8.4 to 3.4) [95% CI, − 1.9 to − 1.6]
Year 1	− 1.3 (− 4.4 to 2.3)	− 1.5 (− 8.4 to 3.0) [95% CI, − 1.7 to − 1.4]
Change	0.2 (− 0.9 to 1.5)	0.2 (− 1.9 to 6.9) [95% CI, 0.1 to 0.3]
Weight		
Baseline	− 1.0 (− 7.7 to 2.5)	− 1.4 (− 10.7 to 2.1) [95% CI, − 1.6 to − 1.3]
Year 1	− 0.6 (− 6.8 to 2.9)	− 0.6 (− 9.0 to 2.5) [95% CI, − 0.8 to − 0.5]
Change	0.4 (− 1.0 to 3.2)	0.8 (− 3.1 to 6.1) [95% CI, 0.7 to 0.9]
Body mass index		
Baseline	0.0 (− 4.9 to 2.4)	− 0.5 (− 4.8 to 3.9) [95% CI, − 0.6 to − 0.3]
Year 1	0.4 (− 3.9 to 3.1)	0.2 (− 5.1 to 3.9) [95% CI, 0.1 to 0.4]
Change	0.4 (− 2.4 to 3.6)	0.6 (− 3.0 to 4.9) [95% CI, 0.5 to 0.7]

*CI* confidence interval

### Comparison of general outcome and efficacy endpoints

There was no graft loss or death in the CRADLE cohort; there was 1 graft loss and 3 deaths in the CERTAIN cohort (Table 3). The rate of BPAR during the 1st year posttransplant was low in both cohorts (5.6% *vs.* 7.8%); the occurrence of the composite efficacy failure endpoint comprising BPAR, graft loss, and death was not significantly different (*P* = 0.33) between the two cohorts (Table 3; Fig. 2b). Mean eGFR at year 1 posttransplant and the degree of proteinuria were also comparable between CRADLE and CERTAIN (Table 3; Fig. 2c).

**Table 3:** Safety parameters, outcome and efficacy endpoints at year 1

Parameters and endpoints	CRADLE control cohort *n* = 54	CERTAIN cohort *n* = 554	*P* value^a^
Graft loss, *n* (%)	0 (0.00)	1 (0.2)	NA
Death, *n* (%)	0 (0.00)	3 (0.5)	NA
Biopsy-proven acute rejection			
Patients with at least one acute rejection episode, *n* (%)	3 (5.6)	43 (7.8)	NA
Number of acute rejection episodes (all), *n*	4	50	NA
Number of only T cell–mediated changes (all), *n* (%)	3 (75.0% of *n* = 4)	40 (80.0% of *n* = 50)	
Number of only antibody-mediated changes (all), *n* (%)	0 (0% of *n* = 4)	6 (12.0% of *n* = 50)	
Number of antibody- and T cell–mediated changes (all), *n* (%)	1 (25.0% of *n* = 4)	4 (8.00% of *n* = 50)	
Occurrence of composite efficacy end point^b^, KM estimate % (SE)	5.8 (3.3)	7.5 (1.1)	0.33
Kidney transplant function			
eGFR, mL/min/1.73 m^2^ (mean ± SD)	72.5 ± 24.9 (*n* = 48)	77.3 ± 24.2 (*n* = 537)	0.19
Proteinuria, g/mol creatinine (mean ± SD)	20.2 ± 13.9 (*n* = 32)	30.6 ± 58.4 (*n* = 76)	0.15
Posttransplant lymphoproliferative disorder, *n* (%)	0 (0.00)	3 (0.5)	NA
Infections			
Patients with at least one infection, *n* (%)	35 (64.8)	354 (63.9)	0.89
Urinary tract infections, *n* (%)	13 (24.1)	86 (15.5)	0.10

^a^*P* values of metric variables were calculated using the pooled *t*-test or the Satterthwaite *t*-test, depending on equality of variance. *P* values for frequency variables were calculated using the chi-squared test. No *P* values were calculated for frequency variables whenever the expected frequency in at least one of the cells was less than 5. The *P* value of the composite endpoint was calculated by comparing the event-free survival rates at 12 months (365 days) posttransplant, using a chi-squared test with one degree of freedom together with the clog test statistic X3 according to Klein et al. ^25^

^b^Composite efficacy endpoint: Composite of BPAR, graft loss or death, whichever came first

*NA* not applicable (no *P* value was calculated, when the expected frequency in the respective contingency table was less than 5)

### Comparison of key safety parameters

No PTLD occurred in the CRADLE cohort, 3 patients (0.5%) developed PTLD in the CERTAIN cohort (Table 3). The number of patients with at least one infection irrespective of the underlying pathogen or site was comparable between the CRADLE cohort and the CERTAIN cohort (Table 3; Fig. 2d). This also applied to the number of patients with at least one urinary tract infection (Table 3; Fig. 2d).

### Comparison of specific laboratory endpoints

We found no significant differences in hemoglobin values, total leukocyte count, and neutrophil count between CRADLE and CERTAIN at year 1 posttransplant (Table 4). The only significant difference was observed for the mean platelet count, which was 13.9% lower in the CRADLE cohort than in the CERTAIN cohort (Table 4).

**Table 4:** Laboratory endpoints of specific interest at year 1

Parameter, mean ± SD	CRADLE control cohort	CERTAIN cohort	*P* value^a^
Hemoglobin [g/dL]	12.3 ± 1.7 (*n* = 38)	12.1 ± 1.5 (*n* = 531)	0.38
Leukocyte [count/nL]	7.1 ± 2.4 (*n* = 37)	7.9 ± 3.4 (*n* = 493)	0.07
Neutrophils [count/nL]	3.6 ± 1.5 (*n* = 39)	3.8 ± 2.2 (*n* = 114)	0.61
Platelets [count/nL]	253 ± 69 (*n* = 39)	294 ± 93 (*n* = 491)	0.001

^a^*P* values of metric variables were calculated using the pooled *t*-test or the Satterthwaite *t*-test, depending on equality of variance

### Data completeness

Supplemental Table 1 compares data completeness at year 1 posttransplant between CRADLE and CERTAIN. Data completeness was higher in CERTAIN regarding eGFR (*P* = 0.0031), hemoglobin level (*P* = 0.0001), leucocyte count (*P* = 0.0001), and platelet count (*P* = 0.0006). Data completeness was higher in CRADLE regarding proteinuria (*P* = 0.0001) and neutrophil count (*P* = 0.0001). The percentage of study completion in CRADLE at year 1 posttransplant was comparable to the percentage of follow-up data available in CERTAIN (*P* = 0.79).

### CERTAIN sub-cohort analyses

The CERTAIN sub-cohort “2012–2016” contained *n* = 266 patients. Supplemental Tables 4–7 show the results obtained from the comparison between the CRADLE control cohort and the CERTAIN sub-cohort “2012–2016.” There was an overall good comparability regarding most baseline patient and transplant characteristics and outcome data at month 12 posttransplant. The following significant differences were noted: There were more living-related donor transplantations (42.6% vs. 27.4%, *P* = 0.03) and more urinary tract infections (24.1% vs. 12.4%, *P* = 0.03) in the CRADLE control cohort than in the CERTAIN sub-cohort 2012–2016. There was a slightly lower leucocyte count (7.1 ± 2.4 vs. 8.1 ± 3.5, *P* = 0.04) and thrombocyte count (253 ± 69 vs. 295 ± 104, *P* = 0.002) in the CRADLE control cohort than in the CERTAIN sub-cohort 2012–2016. The median weight at baseline and the median BMI at baseline and at year 1 posttransplant were higher in the CRADLE control cohort than in the CERTAIN sub-cohort 2012–2016. The change of both weight and BMI was smaller in the CRADLE control cohort than in the CERTAIN sub-cohort 2012–2016 (Supplemental Table 5).

The CERTAIN sub-cohort “basiliximab” contained *n* = 183 patients. Supplemental Tables 8–11 show the results obtained from the comparison between the CRADLE control cohort and the CERTAIN sub-cohort “basiliximab.” This analysis revealed no significant differences regarding most baseline patient and transplant characteristics and outcome data at month 12 posttransplant. The following significant differences were noted: the weight at baseline and the change of weight were smaller in the CRADLE control cohort than in the CERTAIN sub-cohort basiliximab. The BMI at baseline was higher in the CRADLE control cohort than in the CERTAIN sub-cohort basiliximab (Supplemental Table 9).

In the CRADLE control cohort, the rate of patients who remained on the initial immunosuppressive regimen (tacrolimus, MMF and steroids) during the first year posttransplant was higher than in both the CERTAIN sub-cohort 2012–2016 and the CERTAIN sub-cohort basiliximab (see Supplemental Tables 4 and 8).

## Discussion

This is the first study that compares Real-World Data from a specific patient registry with a control cohort from an RCT in pediatric kidney transplantation. Comparative analyses between Real-World Evidence and results derived from RCTs have already been initiated by the FDA, for example as part of the RCT Duplicate project [10]. Although there is not always concordance between Real-World Evidence and respective RCTs, the first results from the RCT Duplicate project, evaluating cardiovascular outcomes of antidiabetic or antiplatelet medications, basically show a good comparability depending on the used agreement metric. Concordance of results is especially enhanced if the respective RCT is emulated as exactly as possible, which is facilitated if the RCT actively compares clearly defined therapy regimens and if design and analysis principles are defined to be exactly reproducible [10]. Accordingly, in our study, we focused on the emulation of the control cohort of the randomized controlled CRADLE trial in pediatric kidney transplantation. The CRADLE trial compares two active therapy regimens and defines inclusion and exclusion criteria specifically, which allowed us to apply a patient attrition model on the Real-World Data derived from CERTAIN as exactly as possible. By that, we were able to generate a CERTAIN cohort as an emulation of the CRADLE control cohort. Both cohorts showed comparable baseline characteristics, outcome and efficacy endpoints relevant to pediatric kidney transplantation, key safety parameters, and laboratory endpoints of specific interest at year 1 posttransplant.

In the CRADLE control, a higher number of patients (87.0%) remained on the initial immunosuppressive regimen (tacrolimus, MMF and steroids) during the first year posttransplant than in the CERTAIN cohort (68.4%). This difference is not unexpected, because patients’ and caregiver’s adherence to a protocol-defined drug regimen within an RCT is higher than under real-world conditions. Accordingly, this difference was also seen in the sensitivity analysis when comparing both the additional CERTAIN sub-cohorts 2012–2016 and basiliximab with the CRADLE control cohort.

Regarding outcome and efficacy parameters, one might expect better patient outcomes in CRADLE compared to the Real-World Data derived from CERTAIN, because RCTs should be associated with a higher, study-specific, follow-up adherence and frequency. Thereby, imminent patient problems might be detected earlier than in Real-World conditions, as reflected by the CERTAIN cohort. However, we observed a comparable mortality and rate of graft loss, a similar rate of BPAR, and a comparable occurrence rate of the composite efficacy failure endpoint comprising BPAR, graft loss, and death. Also, kidney function measured by eGFR at year 1 posttransplant and proteinuria as a marker of transplant damage did not differ between the respective CRADLE and CERTAIN cohorts. Furthermore, even without the neat follow-up conditions as usually provided by RCTs, the Real-World Data represented by the CERTAIN cohort showed the same rate of infections and especially urinary tract infections as reported for the CRADLE control cohort. The observed differences in anthropometric data between CRADLE and CERTAIN were small and not clinically meaningful.

The CERTAIN cohort is ten times as large as the CRADLE control cohort. This comparably larger sample size in CERTAIN allows for a better precision of estimates and detection of rare events. It is noteworthy that the attrition criteria of the CRADLE trial excluded 73% of the patient population of pediatric kidney transplant recipients documented in the CERTAIN registry at the time of data extraction. Hence, patient selection in randomized drug trials might lead to study results and respective conclusions which are only applicable to a small subset of the investigated patient population.

In the CRADLE trial, use of induction therapy with basiliximab was obligatory until February 2013, after which it became optional following a protocol amendment. We therefore did not apply the inclusion criterion “usage of basiliximab” to the CERTAIN cohort in the main analysis. Accordingly, the percentage of patients with basiliximab induction therapy in the CRADLE cohort was higher than in the CERTAIN cohort. Basiliximab induction therapy is not widely used in Europe, because two RCTs in pediatric kidney transplantation have not shown any therapeutic benefit [27, 28]. However, to increase the validity of our results and to support the concept of Real-World Data usage in pediatric kidney transplantation, we generated a basiliximab sub-cohort from the main CERTAIN cohort which emulated the distribution of induction therapy use with basiliximab in the CRADLE control cohort. The comparison of this sub-cohort with the CRADLE control cohort revealed no significant differences in the investigated parameters and endpoints besides the continuation of the initial immunosuppressive regimen at year 1 posttransplant and some anthropometric data, which were small and likely not of clinical relevance.

The proposed approach of Real-World data usage in our study is not consistent with comparing a new intervention with historical cohorts because the latter typically have much smaller patient numbers than do emulated cohorts derived from registry data. In the main analysis, part of the data collection in the CERTAIN registry took place during the same time period at which the CRADLE trial was conducted, thus partly avoiding an era effect. To increase the validity of our results in that respect, we also compared the control cohort of the CRADLE trial with a sub-cohort of the main CERTAIN cohort which contained only patients who received their kidney allograft in the same era as in the CRADLE trial. This analysis also revealed a good comparability to the CRADLE control cohort. The proportion of living-related donors in this CERTAIN sub-cohort (27.4%) was near to the expected frequency of living-donor kidney transplantations in the pediatric population in the USA (31.0%) [13]; however, the difference to CRADLE (42.6% living-donors) was statistically significant. There were small differences in the rate of urinary tract infections, leucocyte count, and thrombocyte count as well as some anthropometric data. However, these differences were small and likely without clinical significance. The slightly lower rate of urinary tract infections in this CERTAIN sub-cohort could be explained by a possible underreporting to the register. The diagnosis of a urinary tract infection in the setting of pediatric kidney transplantation is often made in outpatient settings.

Usage of Real-World Data from CERTAIN to emulate the control cohort of CRADLE has some limitations: (i) Data availability for specific parameters and endpoints differed between CRADLE and CERTAIN. (ii) The number of patients still on study drug regimen (tacrolimus, MMF, steroids) at year 1 posttransplant in CRADLE (87.0%) was higher than in CERTAIN (68.4%). This difference is not unexpected, because patients in an RCT are kept more tightly on a protocol-defined drug regimen than patients under Real-World conditions. (iii) Data entry into a patient registry such as CERTAIN is not as tightly controllable as it is in RCTs. This leads to some degree of uncertainty as to the validity of the Real-World Data and might introduce a systematic bias when comparing the CERTAIN cohort to the randomized controlled CRADLE cohort. Yet, we demonstrate that data completeness in CERTAIN was not inferior compared to the CRADLE trial. (iv) Reasons for drug discontinuation, such as gastroenteritis, are not documented in the minimally required dataset in CERTAIN. (v) Not all endpoints evaluated in the CRADLE trial could be meaningfully compared with the CERTAIN cohort. For example, relevant laboratory read-outs such as donor-specific antibodies against human leukocyte antigens (HLA-DSA) or the rate of viremia of cytomegalovirus, Epstein-Barr virus, or BK polyoma virus could not be compared between the two cohorts because of different methods of measurement in the contributing transplant centers and incomplete documentation in CERTAIN. (vi) Registry data lack standardized diagnostic criteria or equivalent outcome measures and are variable in procedures and duration of follow-up. For example, gastroenterological complications were recorded in less detail or under different terms and definitions in CERTAIN than in CRADLE. (vii) The exact application of study-specific inclusion and exclusion criteria on register data as performed in our analysis is an accepted method to emulate trial data [20]. Besides that, other methods for emulation are currently proposed and performed, for example propensity score matching [10]. We were not able to perform propensity score matching in our analysis, because we had no access to the raw data material of the CRADLE trial. (viii) The FDA proposes the usage of external control cohorts derived from Real-World Data sources in the setting of single-arm clinical trials especially if the expected treatment effect over the control cohort is expected to be large [8]. This specific criterion was not fulfilled by the CRADLE trial. CRADLE showed non-inferiority of the investigated new treatment protocol over standard treatment. However, the FDA-sponsored RCT duplicate project itself also contained other successfully emulated non-inferiority trials [10], which is why we expected before initiating this analysis that the CRADLE trial could be emulated as well.

In conclusion, it was feasible to emulate the control cohort of the CRADLE trial with Real-World Data derived from the CERTAIN Registry. The outcome of children in the CRADLE control cohort might have been expected to be better than in the CERTAIN cohort because of more intensive patient surveillance under the conditions of a controlled clinical study. Counterintuitively, this was not the case in our analysis. Besides a good comparability regarding baseline characteristics, both cohorts also were comparable regarding the vast majority of investigated endpoints. Most of the variables needed to emulate the CRADLE attrition model were available in CERTAIN. Our data suggest that the conduct of single arm trials in the field of pediatric kidney transplantation with external control cohorts generated from good-quality Real-World Data sources such as CERTAIN appears to be feasible in principle. For sure, this might save costs; but more importantly, the representation of the Real-World conditions by control cohorts in clinical trials probably improves their quality, external validity, and generalizability of results to the pediatric kidney transplant patient population in general. The emulation of control cohorts derived from Real-World Data sources might facilitate the conduct of drug trials in pediatric kidney transplantation, if the conduct of an RCT is not feasible, and thereby enhance the time to availability of new drugs in this vulnerable patient population. However, there are potentially relevant sources of bias to consider when using or interpreting Real-World Data from registries such as CERTAIN in the context of clinical trial conduct in pediatric kidney transplantation. Not all relevant data might be documented completely for every patient in a registry, which was specifically the case with donor-specific HLA antibody data in CERTAIN.

## Supplementary Information

Below is the link to the electronic supplementary material.

Supplementary file1 (DOCX 70 KB)

Graphical Abstract (PPTX 172 KB)

## Data Availability

The data that support the findings of this study are available from the corresponding author, [CP], upon reasonable request.

## Abbreviations

BPAR: Biopsy-proven acute rejection
CERTAIN: Cooperative European Paediatric Renal Transplant Initiative
FDA: Food and Drug Administration
HIV: Human immunodeficiency virus
MMF: Mycophenolate mofetil
PTLD: Posttransplant lymphoproliferative disorder
RCT: Randomized controlled trial
