# Supplementary material for: Emulation of the control cohort of a randomized controlled trial in pediatric kidney transplantation with Real-World Data from the CERTAIN Registry
Source: Pediatr Nephrol. 2022 Oct 20;38(5):1621–32. doi: 10.1007/s00467-022-05777-x (PMC9584233; doi:10.1007/s00467-022-05777-x)
Supplement: Supplementary file 1 — Supplementary file1 (DOCX 70 KB) [file 467_2022_5777_MOESM1_ESM.docx]

**SUPPLEMENTAL MATERIAL**

**Contents:**

- Description of the CERTAIN Registry: completeness and quality of data
- Supplemental Table 1: Comparison of data completeness between CRADLE and CERTAIN
- Exclusion criteria of the CRADLE trial
- Exclusion criteria applied to CERTAIN
- Supplemental Table 2: STROBE checklist
- Supplemental Table 3: Exclusion criteria for the CRADLE trial, which were not applicable to the CERTAIN Registry
- Supplemental Table 4. CRADLE control cohort compared to the CERTAIN sub-cohort “2012–2016”. Patient and transplant characteristics.
- Supplemental Table 5. CRADLE control cohort compared to the CERTAIN sub-cohort “2012–2016”. Anthropometric data at baseline and year 1 posttransplant.
- Supplemental Table 6. CRADLE control cohort compared to the CERTAIN sub-cohort “2012–2016”. Safety parameters, outcome and efficacy endpoints at year 1 posttransplant.
- Supplemental Table 7. CRADLE control cohort compared to the CERTAIN sub-cohort “2012–2016”. Laboratory endpoints of specific interest at year 1 posttransplant.
- Supplemental Table 8. CRADLE control cohort compared to the CERTAIN sub-cohort “basiliximab”. Patient and transplant characteristics.
- Supplemental Table 9. CRADLE control cohort compared to the CERTAIN sub-cohort “basiliximab”. Anthropometric data at baseline and year 1 posttransplant.
- Supplemental Table 10. CRADLE control cohort compared to the CERTAIN sub-cohort “basiliximab”. Safety parameters, outcome and efficacy endpoints at year 1 posttransplant.
- Supplemental Table 11. CRADLE control cohort compared to the CERTAIN sub-cohort “basiliximab”. Laboratory endpoints of specific interest at year 1 posttransplant.

**Description of the CERTAIN Registry: completeness and quality of data**

Data entry into the CERTAIN Registry commenced in January 2011. In October 2020, there were 76 centers from 20 countries participating in the registry with 2055 patients enrolled. CERTAIN provides a detailed data capture, thereby allowing an in-depth characterization of specific patient cohorts. Data are registered prior to kidney transplantation, at month 1, 3, 6, and 12 posttransplant and in 6 months intervals thereafter. In addition, the dataset of CERTAIN allows a detailed and flexible documentation of the posttransplant follow-up by continuous entries, which enable documentation of any number of relevant data (e.g., laboratory values, drug therapy). Specific case report forms (CRFs) collect detailed and accurate information on relevant data and events of pediatric kidney transplantation in the peri- and posttransplant course. There are two datasets, the minimally required dataset and the extended dataset. The minimally required dataset is mandatory for all participating centers. The extended dataset provides deeper insight into the patients’ clinical course and treatment by documentation of additional items which are partly predefined and partly definable by the participating center itself.

The CERTAIN web application (accessible via http://www.certain-registry.eu/RegApp) has an automatic and manual data validation functionality. During data entry, the dataset is automatically validated by predefined ranges of plausibility. In addition, the system has an integrated manual quality assurance process. First, documented data require local approval on site; second, a data quality manager at the registry headquarters checks data plausibility randomly. Only data passing this quality assurance process are incorporated into the research database. These functions are active at any time and location, requiring only a common web browser and internet access.

**Supplemental Table 1: Comparison of data completeness between CRADLE and CERTAIN**

|  | | | |  |  |
| --- | --- | --- | --- | --- | --- |
| **Parameters** | | | **CRADLE control cohort, n=54** | **CERTAIN cohort, n=554** | ***P* value** |
|  | | |  |  |  |
| Patients who completed the trial (CRADLE) *vs.* patients documented at year 1 posttransplant (CERTAIN), n (%) | | | 52 (96.3) | 537 (96.9) | 0.79 |
| Patients with available data on kidney transplant function | | |  |  |  |
|  | eGFR, n (%) | | 48 (88.9) | 537 (96.9) | 0.0031 |
|  | Proteinuria, n (%) | | 32 (59.3) | 76 (13.7) | 0.0001 |
|  | | |  |  |  |
| Patients with available data on laboratory endpoints | | |  |  |  |
|  | | Hemoglobin, n (%) | 38 (70.4) | 531 (95.8) | 0.0001 |
|  | | Leukocyte count, n (%) | 37 (68.5) | 493 (88.9) | 0.0001 |
|  | | Neutrophil count, n (%) | 39 (72.2) | 114 (20.6) | 0.0001 |
|  | | Platelet count, n (%) | 39 (72.2) | 491 (88.6) | 0.0006 |

**Exclusion criteria of the CRADLE trial (safety population)**

Key exclusion criteria were nongenetic focal segmental glomerulosclerosis, donor aged >60 years or donors with known kidney disease, donor positive for hepatitis B surface antigen or hepatitis C virus, cold ischemia time >24 hours, multiorgan transplant or previous nonrenal transplant recipients, HLA‐identical living‐related donation, ABO incompatible allograft or positive T‐cell cross‐match, elevated panel reactive antibodies (>20% by a complement-dependent cytotoxicity‐based assay or >50% by a flow cytometry or enzyme-linked immunosorbent-based assay), assessment by the investigator as being at high risk for antibody‐mediated rejection (ABMR) (e.g., presence of preformed donor‐specific antibodies against HLA antigens [HLA‐DSA]), or severe liver disease. At the time of randomization (weeks 4–6 posttransplant), patients were required to be receiving tacrolimus with MMF and steroids, and to have an eGFR >40 mL/min/1.73 m^2^ (updated Schwartz formula^20^). Exclusion criteria at the time of randomization were: treatment for acute rejection two weeks before randomization, steroid-resistant acute rejection, biopsy-proven acute rejection (BPAR) Banff grade ≥IB or ABMR prior to randomization, presence of ongoing wound healing problems, severe surgical complications, nephrotic range proteinuria, severe uncontrolled hypercholesterolemia and recipients with blood count values falling below specified lower limits.^17^ Patients were recruited between August 2012 and October 2016.

**Exclusion criteria applied to CERTAIN**

All patients initially treated with tacrolimus, mycophenolate mofetil and glucocorticoids (steroids) were selected. Patient data were analyzed until month 12 posttransplant, or until the timepoint of graft loss or death, whichever event came first. Patients with an incomplete follow-up for less than 12 months posttransplant due to loss to follow-up were considered. The following exclusion criteria were then applied on the remaining patients: Donors older than 60 years, recipients of an ABO incompatible allograft or with a positive T-cell crossmatch, donors and recipients with a positive serology of human immunodeficiency virus (HIV), hepatitis B surface antigen (for recipients: viral serology results obtained within 6 months prior to transplantation) or hepatitis C, recipients with a history of malignancy, recipients of multiple solid organ transplants, patients with severe liver disease, severe restrictive or obstructive pulmonary disorders, a history of hemolytic uremic syndrome not associated with diarrhea, a severe allergy requiring acute (within 4 weeks of transplantation) or chronic treatment or those with non-genetic focal segmental glomerulosclerosis, patients who required growth hormone therapy prior to transplantation. Figure 1 shows the corresponding patient attrition model. The emulated study cohort based on this attrition model is further referred to as the CERTAIN cohort.

**Supplemental Table 2.** STROBE checklist derived from <https://www.strobe-statement.org/>. This study is based on registry data. Therefore, the following STROBE criteria were not fully applicable to our analysis due to its study design (registry study and emulation of a preexisting clinical trial cohort): Item 6, “Methods/Participants”: not applicable because of our study design. For further information on patient eligibility, see “Description of the CERTAIN Registry: completeness and quality of data” above. Item 7, “Methods/Variables: analysis of confounders and effect modifiers”: not applicable because of our study design. Item 13, (a) and (b), “Results/Participants”: not applicable because of our study design.

|  | Item No | Recommendation |
| --- | --- | --- |
| **Title and abstract** | 1 | (*a*) Indicate the study’s design with a commonly used term in the title or the abstract |
|  |  | (*b*) Provide in the abstract an informative and balanced summary of what was done and what was found |
| Introduction | | |
| Background/rationale | 2 | Explain the scientific background and rationale for the investigation being reported |
| Objectives | 3 | State specific objectives, including any prespecified hypotheses |
| Methods | | |
| Study design | 4 | Present key elements of study design early in the paper |
| Setting | 5 | Describe the setting, locations, and relevant dates, including periods of recruitment, exposure, follow-up, and data collection |
| Participants | 6 | (*a*) *Cohort study*—Give the eligibility criteria, and the sources and methods of selection of participants. Describe methods of follow-up  *Case-control study*—Give the eligibility criteria, and the sources and methods of case ascertainment and control selection. Give the rationale for the choice of cases and controls  *Cross-sectional study*—Give the eligibility criteria, and the sources and methods of selection of participants |
|  |  | (*b*) *Cohort study*—For matched studies, give matching criteria and number of exposed and unexposed  *Case-control study*—For matched studies, give matching criteria and the number of controls per case |
| Variables | 7 | Clearly define all outcomes, exposures, predictors, potential confounders, and effect modifiers. Give diagnostic criteria, if applicable |
| Data sources/ measurement | 8* | For each variable of interest, give sources of data and details of methods of assessment (measurement). Describe comparability of assessment methods if there is more than one group |
| Bias | 9 | Describe any efforts to address potential sources of bias |
| Study size | 10 | Explain how the study size was arrived at |
| Quantitative variables | 11 | Explain how quantitative variables were handled in the analyses. If applicable, describe which groupings were chosen and why |
| Statistical methods | 12 | (*a*) Describe all statistical methods, including those used to control for confounding |
|  |  | (*b*) Describe any methods used to examine subgroups and interactions |
|  |  | (*c*) Explain how missing data were addressed |
|  |  | (*d*) *Cohort study*—If applicable, explain how loss to follow-up was addressed  *Case-control study*—If applicable, explain how matching of cases and controls was addressed  *Cross-sectional study*—If applicable, describe analytical methods taking account of sampling strategy |
|  |  | (*e*) Describe any sensitivity analyses |
| Results | | |
| Participants | 13* | (a) Report numbers of individuals at each stage of study—eg numbers potentially eligible, examined for eligibility, confirmed eligible, included in the study, completing follow-up, and analysed |
|  |  | (b) Give reasons for non-participation at each stage |
|  |  | (c) Consider use of a flow diagram |
| Descriptive data | 14* | (a) Give characteristics of study participants (eg demographic, clinical, social) and information on exposures and potential confounders |
|  |  | (b) Indicate number of participants with missing data for each variable of interest |
|  |  | (c) *Cohort study*—Summarise follow-up time (eg, average and total amount) |
| Outcome data | 15* | *Cohort study*—Report numbers of outcome events or summary measures over time |
|  |  | *Case-control study—*Report numbers in each exposure category, or summary measures of exposure |
|  |  | *Cross-sectional study—*Report numbers of outcome events or summary measures |
| Main results | 16 | (*a*) Give unadjusted estimates and, if applicable, confounder-adjusted estimates and their precision (eg, 95% confidence interval). Make clear which confounders were adjusted for and why they were included |
|  |  | (*b*) Report category boundaries when continuous variables were categorized |
|  |  | (*c*) If relevant, consider translating estimates of relative risk into absolute risk for a meaningful time period |
| Other analyses | 17 | Report other analyses done—eg analyses of subgroups and interactions, and sensitivity analyses |
| Discussion | | |
| Key results | 18 | Summarise key results with reference to study objectives |
| Limitations | 19 | Discuss limitations of the study, taking into account sources of potential bias or imprecision. Discuss both direction and magnitude of any potential bias |
| Interpretation | 20 | Give a cautious overall interpretation of results considering objectives, limitations, multiplicity of analyses, results from similar studies, and other relevant evidence |
| Generalisability | 21 | Discuss the generalisability (external validity) of the study results |
| Other information | | |
| Funding | 22 | Give the source of funding and the role of the funders for the present study and, if applicable, for the original study on which the present article is based |

**Supplemental Table 3.** Exclusion criteria for the CRADLE trial, which were not applicable to the CERTAIN Registry

| **Criteria** | **Reasons for non-application** |
| --- | --- |
| Recipients of kidneys from donors with known kidney disease (such as diabetic nephropathy or nephrosclerosis) at the time of transplantation. | Donor kidney disease not documented in CERTAIN. |
| Recipients with allografts from HLA-identical living related donors. | Pediatric kidney transplant recipients are not eligible for HLA-identical living related donors (minor twins). |
| Pregnant or nursing (lactating) female recipients. | Not documented in CERTAIN |
| Recipients considered at high risk of antibody mediated acute rejection (e.g. presence of pre-formed DSA, if known). | The information on pre-formed DSA is available in CERTAIN only for a subset of patients. Instead, we applied the exclusion criterion “panel-reactive antibodies (PRA) >20%”. |

| **Supplemental Table 4. CRADLE control cohort compared to the CERTAIN sub-cohort “2012-2016”. Patient and transplant characteristics.** | | | | |
| --- | --- | --- | --- | --- |
| **Patient characteristics** | | **CRADLE control cohort**  **n=54** | **CERTAIN sub-cohort “2012-2016”**  **n=266** | ***P* Value**^1^ |
|  | |  |  |  |
| Age at KTx (years), mean ± SD | | 10.3 ± 4.8 | 10.7 ± 4.6 | 0.56 |
| Male sex, n (%) | | 31 (57.4) | 153 (57.5) | 0.99 |
| White/Caucasian race, n (%) | | 47 (87.0) | 249 (93.6) | NA |
| Diabetes mellitus at randomization, n (%) | | 2 (3.7) | 1 (0.4) | NA |
|  | |  |  |  |
| **Primary kidney diseases, n (%)** | |  |  | NA |
|  | Renal hypoplasia/dysplasia | 19 (35.2) | 61 (22.9) |  |
|  | Obstructive uropathy/vesicoureteral reflux | 7 (13.0) | 47 (17.7) |  |
|  | Polycystic kidney disease | 5 (9.3) | 38 (14.3) |  |
|  | Glomerulonephritis/glomerular disease | 12 (22.2) | 47 (17.7) |  |
|  | Systemic disorders including vasculitis | 2 (3.7) | 21 (7.9) |  |
|  | Tubular disorder | 3 (5.6) | 1 (0.4) |  |
|  | Other | 6 (11.1) | 51 (19.2) |  |
|  |  |  |  |  |
| **Immunosuppressants at KTx, n (%)** | |  |  |  |
|  | Induction therapy (basiliximab or daclizumab) | 50 (92.6) | 96 (36.1) | <0.0001 |
|  | Tacrolimus + MMF + Steroids | 54 (100) | 264 (99.3) | NA |
|  |  |  |  |  |
| **Immunosuppressants at year 1 posttransplant, n (%)** | |  |  |  |
|  | Tacrolimus + MMF + steroids | 47 (87.0)^2^ | 170 (63.9) | 0.0009 |
|  | |  |  |  |
| **Renal replacement therapy at time of KTx** | |  |  | 0.16 |
|  | None (preemptive KTx) | 21 (38.9) | 69 (26.0) |  |
|  | Hemodialysis | 16 (29.6) | 95 (35.7) |  |
|  | Peritoneal dialysis | 17 (31.5) | 102 (38.3) |  |
|  |  |  |  |  |
| **Donor-related data** | |  |  |  |
|  | Age, mean ± SD | 27.5 ± 15.0 | 24.9 ± 16.8 | 0.29 |
|  | Living-related donor | 23 (42.6) | 73 (27.4) | 0.03 |
|  |  |  |  |  |
| **Transplant function** | |  |  |  |
|  | Delayed graft function, n (%) | 2 (3.7) | 22 (8.3) | NA |
|  |  |  |  |  |
| ^1^*P* values of metric variables were calculated using the pooled t-test or the Satterthwaite t-test, depending on equality of variance. *P* values for frequency variables were calculated using the chi-squared test. No *p* values were calculated for frequency variables whenever the expected frequency in at least one of the cells was less than five. ^2^According to Supplemental Table 1 of the CRADLE trial.^18^  *MMF, Mycophenolate mofetil; KTx, kidney transplantation, NA, not applicable (no P value was calculated, when the expected frequency in the respective contingency table was less than 5).* | | | | |

| **Supplemental Table 5. CRADLE control cohort compared to the CERTAIN sub-cohort “2012–2016”. Anthropometric data at baseline and year 1 posttransplant.** | | | | | |
| --- | --- | --- | --- | --- | --- |
| **Z-scores, median (range)** | | | **CRADLE control cohort**  **n=54** | **CERTAIN sub-cohort “2012–2016”**  **baseline, n=266**  **year 1, n=256** | |
|  | |  |  |  | |
| **Height** | |  |  |  | |
|  | Baseline | | –1.5 (–4.5 to 2.2) | –1.7 (–7.2 to 3.4)  [95% CI, –1.9 to –1.5] | |
|  | Year 1 | | –1.3 (–4.4 to 2.3) | –1.5 (–7.5 to 3.0)  [95% CI, –1.7 to –1.3] | |
|  | Change | | 0.2 (–0.9 to 1.5) | 0.2 (–1.9 to 5.1)  [95% CI, 0.1 to 0.3] | |
| **Weight** | |  |  |  | |
|  | Baseline | | –1.0 (–7.7 to 2.5) | –1.5 (–10.7 to 2.1)  [95% CI, –1.7 to –1.3] | |
|  | Year 1 | | –0.6 (–6.8 to 2.9) | –0.7 (–9.0 to 2.2)  [95% CI, –0.9 to –0.5] | |
|  | Change | | 0.4 (–1.0 to 3.2) | 0.8 (–2.8 to 6.1)  [95% CI, 0.7 to 0.9] | |
| **Body mass index** | |  |  |  | |
|  | Baseline | | 0.0 (–4.9 to 2.4) | –0.6 (–4.8 to 2.5)  [95% CI, –0.7 to –0.3] | |
|  | Year 1 | | 0.4 (–3.9 to 3.1) | 0.2 (–5.1 to 3.9)  [95% CI, 0.0 to 0.3] | |
|  | Change | | 0.4 (–2.4 to 3.6) | 0.6 (–3.0 to 4.9)  [95% CI, 0.5 to 0.7] | |
|  | |  |  |  |  |
| *CI, confidence interval* | | | | | |

| **Supplemental Table 6. CRADLE control cohort compared to the CERTAIN sub-cohort “2012–2016”. Safety parameters, outcome and efficacy endpoints at year 1 posttransplant.** | | | | | |
| --- | --- | --- | --- | --- | --- |
| **Parameters and Endpoints** | | | **CRADLE control cohort**  **n=54** | **CERTAIN sub-cohort “2012–2016”**  **n=266** | ***P* Value**^1^ |
|  | | |  |  |  |
| **Graft loss, n (%)** | | | 0 (0.00) | 1 (0.38) | NA |
| **Death, n (%)** | | | 0 (0.00) | 2 (0.75) | NA |
|  | | |  |  |  |
| **Biopsy-proven acute rejection** | | |  |  |  |
|  | Patients with at least one acute rejection episode, n (%) | | 3 (5.6) | 17 (6.4) | NA |
|  | Number of acute rejection episodes (all), n | | 4 | 19 | NA |
|  | Number of only T cell-mediated changes (all), n (%) | | 3 (75.0% of n=4) | 13 (68.4% of n=19) |  |
|  | Number of only antibody-mediated changes (all), n (%) | | 0 (0% of n=4) | 4 (21.1% of n=19) |  |
|  | Number of antibody- and T cell-mediated changes (all), n (%) | | 1 (25.0% of n=4) | 2 (10.5% of n=19) |  |
|  |  | |  |  |  |
| **Occurrence of composite efficacy end point^2^, KM estimate % (SE)** | | | 5.8 (3.3) | 6.4 (1.5) | 0.13 |
|  | | |  |  |  |
| **Renal transplant function** | | |  |  |  |
|  | eGFR, mL/min/1.73 m^2^ (mean ± SD) | | 72.5 ± 24.9  (n=48) | 77.7 ± 24.2  (n=256) | 0.18 |
|  | Proteinuria, g/mol creatinine (mean ± SD) | | 20.2 ± 13.9  (n=32) | 22.6 ± 26.3  (n=35) | 0.64 |
|  |  | |  |  |  |
| **Posttransplant lymphoproliferative disorder, n (%)** | | | 0 (0.00) | 1 (0.38) | NA |
|  | | |  |  |  |
| **Infections** | | |  |  |  |
|  | Patients with at least one infection, n (%) | | 35 (64.8) | 165 (62.0) | 0.70 |
|  | Urinary tract infections, n (%) | | 13 (24.1) | 33 (12.4) | 0.03 |
|  |  |  |  |  |  |
|  |  | |  |  |  |
| ^1^*P* values of metric variables were calculated using the pooled T-test or the Satterthwaite t-test, depending on equality of variance. *P* values for frequency variables were calculated using the chi-squared test. No *P* values were calculated for frequency variables whenever the expected frequency in at least one of the cells was less than five. The *P* value of the composite endpoint was calculated by comparing the event-free survival rates at 12 months (365 days) posttransplant, using a chi-squared test with one degree of freedom together with the clog test statistic X3 according to Klein et al, 2007.^26^  ^2^Composite efficacy endpoint: Composite of BPAR, graft loss or death, whichever came first  *NA, not applicable (no P value was calculated, when the expected frequency in the respective contingency table was less than 5).* | | | | | |

| **Supplemental Table 7. CRADLE control cohort compared to the CERTAIN sub-cohort “2012–2016”. Laboratory endpoints of specific interest at year 1 posttransplant.** | | | |
| --- | --- | --- | --- |
| **Parameter, mean ± SD** | **CRADLE control cohort** | **CERTAIN sub-cohort “2012–2016”** | ***P* Value**^1^ |
|  |  |  |  |
| Hemoglobin [g/dL] | 12.3 ± 1.7  (n=38) | 12.1 ± 1.5  (n=252) | 0.47 |
| Leukocyte [count/nL] | 7.1 ± 2.4  (n=37) | 8.1 ± 3.5  (n=238) | 0.04 |
| Neutrophils [count/nL] | 3.6 ± 1.5  (n=39) | 3.9 ± 2.9  (n=52) | 0.58 |
| Platelets [count/nL] | 253 ± 69  (n=39) | 295 ± 104  (n=237) | 0.002 |
|  |  |  |  |
| ^1^*P* values of metric variables were calculated using the pooled t-test or the Satterthwaite t-test, depending on equality of variance. | | | |

| **Supplemental Table 8. CRADLE control cohort compared to the CERTAIN sub-cohort “basiliximab”. Patient and transplant characteristics.** | | | | |
| --- | --- | --- | --- | --- |
| **Patient characteristics** | | **CRADLE control cohort**  **n=54** | **CERTAIN sub-cohort “basiliximab”**  **n=183** | ***P* Value**^1^ |
|  | |  |  |  |
| Age at KTx (years), mean ± SD | | 10.3 ± 4.8 | 11.0 ± 4.6 | 0.32 |
| Male sex, n (%) | | 31 (57.4) | 97 (53.0) | 0.57 |
| White/Caucasian race, n (%) | | 47 (87.0) | 163 (89.1) | 0.68 |
| Diabetes mellitus at randomization, n (%) | | 2 (3.7) | 0 (0.0) | NA |
|  | |  |  |  |
| **Primary kidney diseases, n (%)** | |  |  | NA |
|  | Renal hypoplasia/dysplasia | 19 (35.2) | 27 (14.8) |  |
|  | Obstructive uropathy/vesicoureteral reflux | 7 (13.0) | 34 (18.6) |  |
|  | Polycystic kidney disease | 5 (9.3) | 25 (13.7) |  |
|  | Glomerulonephritis/glomerular disease | 12 (22.2) | 38 (20.8) |  |
|  | Systemic disorders including vasculitis | 2 (3.7) | 11 (6.0) |  |
|  | Tubular disorder | 3 (5.6) | 5 (2.7) |  |
|  | Other | 6 (11.1) | 43 (23.5) |  |
|  |  |  |  |  |
| **Immunosuppressants at KTx, n (%)** | |  |  |  |
|  | Induction therapy (basiliximab or daclizumab) | 50 (92.6) | 169 (92.4) | NA |
|  | Tacrolimus + MMF + Steroids | 54 (100) | 182 (99.5) | NA |
|  |  |  |  |  |
| **Immunosuppressants at year 1 posttransplant, n (%)** | |  |  |  |
|  | Tacrolimus + MMF + steroids | 47 (87.0)^2^ | 115 (62.8) | 0.0008 |
|  | |  |  |  |
| **Renal replacement therapy at time of KTx** | |  |  | 0.29 |
|  | None (preemptive KTx) | 21 (38.9) | 51 (27.9) |  |
|  | Hemodialysis | 16 (29.6) | 60 (32.8) |  |
|  | Peritoneal dialysis | 17 (31.5) | 72 (39.3) |  |
|  |  |  |  |  |
| **Donor-related data** | |  |  |  |
|  | Age, mean ± SD | 27.5 ± 15.0 | 27.4 ± 17.8 | 0.96 |
|  | Living-related donor | 23 (42.6) | 65 (35.5) | 0.34 |
|  |  |  |  |  |
| **Transplant function** | |  |  |  |
|  | Delayed graft function, n (%) | 2 (3.7) | 7 (3.83) | NA |
|  |  |  |  |  |
| ^1^*P* values of metric variables were calculated using the pooled t-test or the Satterthwaite t-test, depending on equality of variance. *P* values for frequency variables were calculated using the chi-squared test. No *p* values were calculated for frequency variables whenever the expected frequency in at least one of the cells was less than five. ^2^According to Supplemental Table 1 of the CRADLE trial.^18^  *MMF, Mycophenolate mofetil; KTx, kidney transplantation, NA, not applicable (no P value was calculated, when the expected frequency in the respective contingency table was less than 5).* | | | | |

| **Supplemental Table 9. CRADLE control cohort compared to the CERTAIN sub-cohort “basiliximab”. Anthropometric data at baseline and year 1 posttransplant.** | | | | | |
| --- | --- | --- | --- | --- | --- |
| **Z-scores, median (range)** | | | **CRADLE control cohort**  **n=54** | **CERTAIN sub-cohort “basiliximab”**  **baseline, n=183  year 1, n=178** | |
|  | |  |  |  | |
| **Height** | |  |  |  | |
|  | Baseline | | –1.5 (–4.5 to 2.2) | –1.8 (–8.4 to 2.4)  [95% CI, –2.1 to –1.5] | |
|  | Year 1 | | –1.3 (–4.4 to 2.3) | –1.5 (–8.4 to 3.0)  [95% CI, –1.9 to –1.2] | |
|  | Change | | 0.2 (–0.9 to 1.5) | 0.2 (–1.0 to 6.9)  [95% CI, 0.1 to 0.3] | |
| **Weight** | |  |  |  | |
|  | Baseline | | –1.0 (–7.7 to 2.5) | –1.4 (–10.7 to 1.9)  [95% CI, –1.6 to –1.1] | |
|  | Year 1 | | –0.6 (–6.8 to 2.9) | –0.6 (–9.0 to 2.0)  [95% CI, –0.9 to –0.4] | |
|  | Change | | 0.4 (–1.0 to 3.2) | 0.7 (–3.1 to 6.1)  [95% CI, 0.5 to 0.8] | |
| **Body mass index** | |  |  |  | |
|  | Baseline | | 0.0 (–4.9 to 2.4) | –0.4 (–4.5 to 3.9)  [95% CI, –0.5 to –0.2] | |
|  | Year 1 | | 0.4 (–3.9 to 3.1) | 0.3 (–5.1 to 3.9)  [95% CI, 0.0 to 0.4] | |
|  | Change | | 0.4 (–2.4 to 3.6) | 0.5 (–2.5 to 4.9)  [95% CI, 0.4 to 0.7] | |
|  | |  |  |  |  |
| *CI, confidence interval* | | | | | |

| **Supplemental Table 10. CRADLE control cohort compared to the CERTAIN sub-cohort “basiliximab”. Safety parameters, outcome and efficacy endpoints at year 1 posttransplant.** | | | | | |
| --- | --- | --- | --- | --- | --- |
| **Parameters and Endpoints** | | | **CRADLE control cohort**  **n=54** | **CERTAIN sub-cohort “basiliximab”**  **n=183** | ***P* Value**^1^ |
|  | | |  |  |  |
| **Graft loss, n (%)** | | | 0 (0.00) | 0 (0.00) | NA |
| **Death, n (%)** | | | 0 (0.00) | 0 (0.00) | NA |
|  | | |  |  |  |
| **Biopsy-proven acute rejection** | | |  |  |  |
|  | Patients with at least one acute rejection episode, n (%) | | 3 (5.6) | 10 (5.5) | NA |
|  | Number of acute rejection episodes (all), n | | 4 | 12 | NA |
|  | Number of only T cell-mediated changes (all), n (%) | | 3 (75.0% of n=4) | 10 (83.3% of n=12) |  |
|  | Number of only antibody-mediated changes (all), n (%) | | 0 (0% of n=4) | 2 (16.7% of n=12) |  |
|  | Number of antibody- and T cell-mediated changes (all), n (%) | | 1 (25.0% of n=4) | 0 (0.00% of n=12) |  |
|  |  | |  |  |  |
| **Occurrence of composite efficacy end point^2^, KM estimate % (SE)** | | | 5.8 (3.3) | 5.0 (1.6) | 0.19 |
|  | | |  |  |  |
| **Renal transplant function** | | |  |  |  |
|  | eGFR, mL/min/1.73 m^2^ (mean ± SD) | | 72.5 ± 24.9  (n=48) | 76.5 ± 22.7  (n=178) | 0.29 |
|  | Proteinuria, g/mol creatinine (mean ± SD) | | 20.2 ± 13.9  (n=32) | 23.0 ± 29.3  (n=23) | 0.68 |
|  |  | |  |  |  |
| **Posttransplant lymphoproliferative disorder, n (%)** | | | 0 (0.00) | 0 (0.0) | NA |
|  | | |  |  |  |
| **Infections** | | |  |  |  |
|  | Patients with at least one infection, n (%) | | 35 (64.8) | 118 (64.5) | 0.96 |
|  | Urinary tract infections, n (%) | | 13 (24.1) | 29 (15.9) | 0.16 |
|  |  |  |  |  |  |
|  |  | |  |  |  |
| ^1^*P* values of metric variables were calculated using the pooled T-test or the Satterthwaite t-test, depending on equality of variance. *P* values for frequency variables were calculated using the chi-squared test. No *P* values were calculated for frequency variables whenever the expected frequency in at least one of the cells was less than five. The *P* value of the composite endpoint was calculated by comparing the event-free survival rates at 12 months (365 days) posttransplant, using a chi-squared test with one degree of freedom together with the clog test statistic X3 according to Klein et al, 2007.^26^  ^2^Composite efficacy endpoint: Composite of BPAR, graft loss or death, whichever came first  *NA, not applicable (no P value was calculated, when the expected frequency in the respective contingency table was less than 5).* | | | | | |

| **Supplemental Table 11. CRADLE control cohort compared to the CERTAIN sub-cohort “basiliximab”. Laboratory endpoints of specific interest at year 1 posttransplant.** | | | |
| --- | --- | --- | --- |
| **Parameter, mean ± SD** | **CRADLE control cohort** | **CERTAIN sub-cohort “basiliximab”** | ***P* Value**^1^ |
|  |  |  |  |
| Hemoglobin [g/dL] | 12.3 ± 1.7  (n=38) | 12.2 ± 1.5  (n=175) | 0.66 |
| Leukocyte [count/nL] | 7.1 ± 2.4  (n=37) | 7.5 ± 2.4  (n=153) | 0.40 |
| Neutrophils [count/nL] | 3.6 ± 1.5  (n=39) | 3.7 ± 2.3  (n=47) | 0.84 |
| Platelets [count/nL] | 253 ± 69  (n=39) | 279 ± 90  (n=151) | 0.09 |
|  |  |  |  |
| ^1^*P* values of metric variables were calculated using the pooled t-test or the Satterthwaite t-test, depending on equality of variance. | | | |
