# Supplementary material for: Emulation of the control cohort of a randomized controlled trial in pediatric kidney transplantation with Real-World Data from the CERTAIN Registry
Source: Pediatr Nephrol. 2022 Oct 20;38(5):1621–32. doi: 10.1007/s00467-022-05777-x (PMC9584233; doi:10.1007/s00467-022-05777-x)
Supplement: Supplementary file 2 — Graphical Abstract (PPTX 172 KB) [file 467_2022_5777_MOESM2_ESM.pptx]

## Slide 1
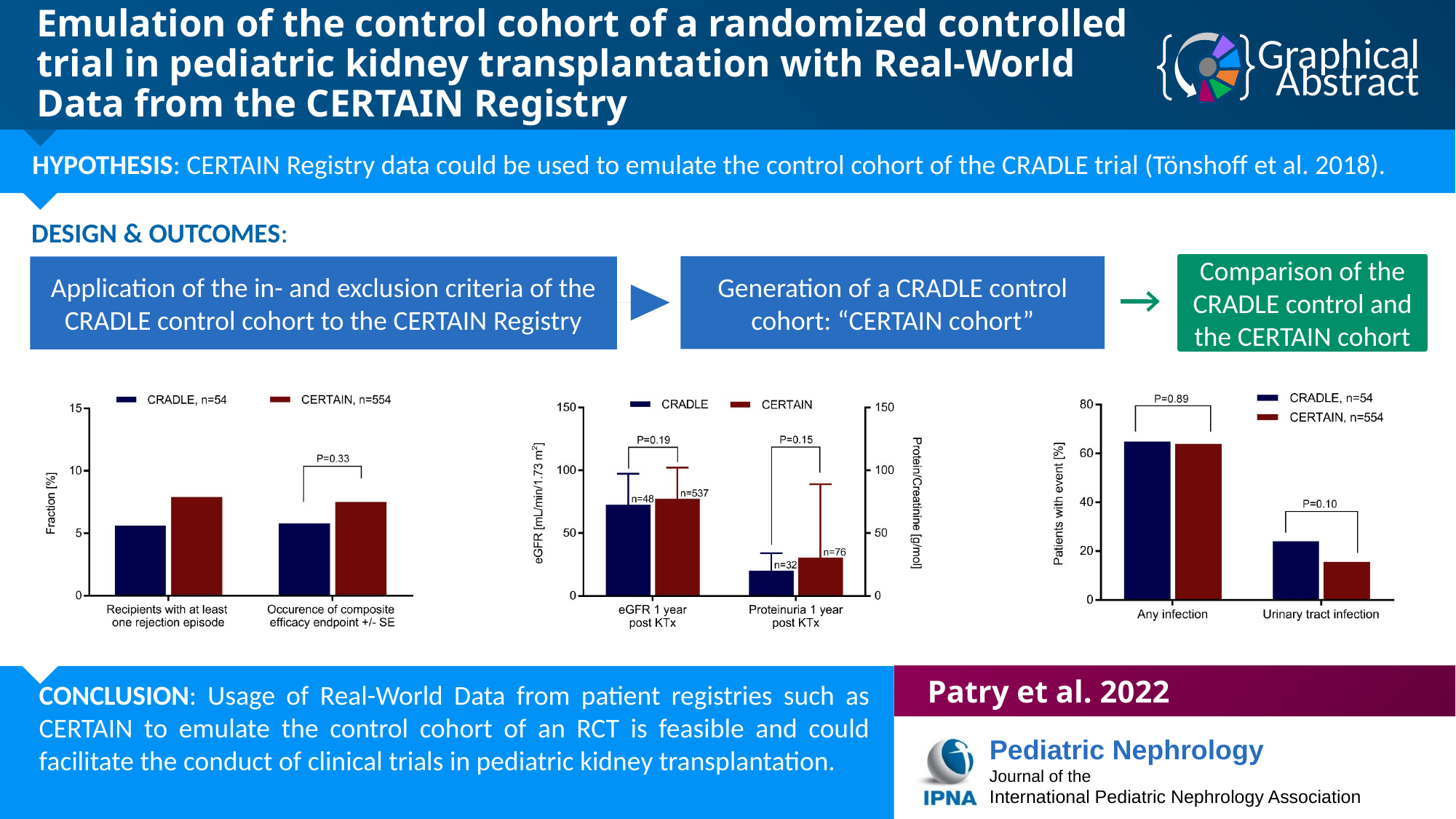

Emulation of the control cohort of a randomized controlled trial in pediatric kidney transplantation with Real-World Data from the CERTAIN Registry
HYPOTHESIS: CERTAIN Registry data could be used to emulate the control cohort of the CRADLE trial (Tönshoff et al. 2018).
DESIGN & OUTCOMES:
Generation of a CRADLE control cohort: “CERTAIN cohort”
Comparison of the CRADLE control and the CERTAIN cohort
Application of the in- and exclusion criteria of the CRADLE control cohort to the CERTAIN Registry
Patry et al. 2022
CONCLUSION: Usage of Real-World Data from patient registries such as CERTAIN to emulate the control cohort of an RCT is feasible and could facilitate the conduct of clinical trials in pediatric kidney transplantation.
